# Rethinking health, human rights, and the climate crisis in the new global order

**DOI:** 10.1093/eurpub/ckag039

**Published:** 2026-06-11

**Authors:** David W Patterson

**Affiliations:** Groningen Centre for Health Law, Faculty of Law, University of Groningen, Groningen, The Netherlands

We are all in a leaking boat in increasingly stormy seas. Some of us are strong swimmers or have (economic) lifejackets. Others can’t swim or are young, differently abled, old or infirm. We share our common humanity but speak different languages. Do we fix our boat together, or do we allow the privileged to abandon ship, leaving many to drown? In our warming and increasingly unstable world, this viewpoint argues for strengthened legal literacy among the public health community, decolonized engagement with Global South partners, and respect for Indigenous knowledge in stewarding our planet.

We have been here before. Recall the two world wars and global chaos which led to the modern human rights movement. The right to health has been with us since the adoption of the WHO Constitution in 1946 and the Universal Declaration of Human Rights in 1948. Today, climate change is the greatest certain and immediate threat to human health and life [1]. Unchecked, it will undermine all other global health efforts.

When climate leadership is most needed, the USA has left the WHO and the Paris Agreement and is leaving the 1992 UN Framework Convention on Climate Change, along with dozens of other multilateral development frameworks, agencies and forums. The USA has never recognized health as a legal right, domestically or internationally. USA support for global health was always equivocal. Certainly, until 2025 the USA supported global health programmes and institutions such as WHO, UNAIDS and the Global Fund to Fight AIDS, Tuberculosis and Malaria, technically and financially. Even so, national economic interests prevailed. In developing guidance on noncommunicable diseases, the USA rejected the science-informed WHO ‘best buy’ on taxes on sugar-sweetened beverages. While the USA initially supported the Pandemic Agreement, it walked away from the draft after limiting financial commitments and emphasizing national sovereignty and private intellectual property rights. Given the wrecking ball of the Trump administration, some ask if the USA departure is indeed a good thing. We can hope and plan for collaboration with the USA under a more moderate administration. But right now, European countries and other middle powers must rally to defend and strengthen science-informed, human rights-based approaches to global health and its determinants.

This includes strengthening institutions and programmes to reflect the obligations owed to Global South States. Indeed, international law affirms these obligations. In July 2025, the International Court of Justice rebuffed the USA, Saudi Arabia, Australia and other fossil fuel exporting states which put short-term national economic interests ahead of global health [2]. These states had argued that their Paris Agreement commitments to address the climate crisis excluded more general human rights obligations. Instead, the world’s top court affirmed that treaty and customary human rights law–including obligations to protect and promote the right to health–applies to all UN Member States.

Regional human rights frameworks and declarations also affirm the right to health, directly or indirectly, in Africa, the Americas, the Arab States, Asia, and Europe. So do the constitutions of over 100 states [3]. These UN and regional legal frameworks have long informed national and regional health policies and the decisions of national courts. Within the European Union, the EU Global Health Strategy 2022–2030 explicitly positions global health as an essential pillar of the EU’s external policy, guided by fundamental values including solidarity, equity, and the respect of human rights. The EU Action Plan on Human Rights and Democracy reinforces these commitments, making it a legal and policy mandate for all EU external action.

These obligations must guide Europe’s national and international development assistance and cooperation. The EU Global Health Strategy 2022–2030 envisages expanded partnerships with actors in other regions, emphasizing co-ownership and mutual interest (Guiding Principle 17). In practice this should include exchanges between Europe and Global South States of both early-career and top researchers and practitioners for the cross-fertilization of ideas, knowledge and skills [4].

Commitments to rights-based approaches to health are hard-won and fragile. Even within the public health community, the right to health is sometimes framed as an exclusively ethical concept–overlooking its power as a legal right. Of course, the legal right to health is not a right to be healthy, which would be nonsense. It is time for the public health community to strengthen its rights literacy, moving beyond expressions of empathy and solidarity which have no legal force. We have the tools to repair our leaking boat. Let’s use them.

In facing the triple planetary crises of climate change, biodiversity loss and pollution, we should be guided by Indigenous Peoples, who have stewarded fragile environments for millennia. Participation of affected communities is a key tenet of the human rights-based approach. In practice this includes structured community representation and participation in the planning and delivery of health services and in addressing the social and legal determinants of health. The COVID-19 pandemic revealed that civil preparedness for epidemics was weak in many countries. Communities did not understand public health measures needed to limit the spread and impact of deadly infections. Technical solutions by way of vaccines faltered where understanding and trust were lacking. Conversely, freedoms of speech, association and assembly are also essential to the right to health. Everyone must be free to advocate for public health priorities, including climate action and the right to a healthy environment. This includes the right to peaceful protest [5].

Together we are sailing into more extreme weather and sea level rise. The liveable planetary ‘human climate niche’ is shrinking. Climate change is bringing crop failures, rising food prices, mass migration and increased conflicts and war. Geophysical tipping points loom. For eight decades the Universal Declaration of Human Rights and international human rights law have charted an ethical and legal course towards better health for all. Today we have left calmer waters but can avoid the worst if we act, together, now. Our boat is leaking, but it is not yet sinking.

Conflict of interest: The author declares no conflicts of interest arise in this Viewpoint.

## Data Availability

No new data were generated or analysed in support of this research.
